# Thermophysical and Mechanical Properties of Hardened Cement Paste with Microencapsulated Phase Change Materials for Energy Storage

**DOI:** 10.3390/ma7128070

**Published:** 2014-12-16

**Authors:** Hongzhi Cui, Wenyu Liao, Shazim Ali Memon, Biqin Dong, Waiching Tang

**Affiliations:** 1 Guangdong Provincial Key Laboratory of Durability for Marine Civil Engineering, College of Civil Engineering, Shenzhen University, Shenzhen 518060, China; E-Mails: h.z.cui@szu.edu.cn (H.C.); dongbq@gmail.com (B.D.); 2 Department of Civil Engineering, COMSATS Institute of Information Technology, Abbottabad Campus, Abbottabad 22010, Pakistan; E-Mail: shazimalimemon@gmail.com; 3 School of Architecture and Built Environment, the University of Newcastle, Callaghan, NSW 2308, Australia; E-Mail: patrick.tang@newcastle.edu.au

**Keywords:** microencapsulated phase change material (MPCM), cement-based materials, thermal energy storage, mechanical properties, thermal properties

## Abstract

In this research, structural-functional integrated cement-based materials were prepared by employing cement paste and a microencapsulated phase change material (MPCM) manufactured using urea-formaldehyde resin as the shell and paraffin as the core material. The encapsulation ratio of the MPCM could reach up to 91.21 wt%. Thermal energy storage cement pastes (TESCPs) incorporated with different MPCM contents (5%, 10%, 15%, 20% and 25% by weight of cement) were developed, and their thermal and mechanical properties were studied. The results showed that the total energy storage capacity of the hardened cement specimens with MPCM increased by up to 3.9-times compared with that of the control cement paste. The thermal conductivity at different temperature levels (35–36 °C, 55–56 °C and 72–74 °C) decreased with the increase of MPCM content, and the decrease was the highest when the temperature level was 55–56 °C. Moreover, the compressive strength, flexural strength and density of hardened cement paste decreased with the increase in MPCM content linearly. Among the evaluated properties, the compressive strength of TESCPs had a larger and faster degradation with the increase of MPCM content.

## 1. Introduction

The building sector is the dominant energy consumer with a 40% share of the overall energy consumption in the world [1,2] and is also responsible for one-third of the greenhouse gas emissions around the world [3]. Moreover, in recent years, the energy demand for buildings has increased very rapidly due to population growth, enhancement of building services and thermal comfort levels, as well as the increase in the time that people spend inside buildings [4]. Furthermore, it is predicted that fossil fuels will continue to produce 75%–80% of the world’s primary energy by 2030 [5]. Thus, the increase in energy demand, the shortage of fossil fuels and environmental concerns have provided the impetus for the development of sustainable building and renewable energy resources. One of the technologies is the thermal energy storage method, which has been considered a simple and effective technique to enhance the energy efficiency of buildings. This, in turn, reduces the environmental impact related to energy use [6].

**Figure 1:**
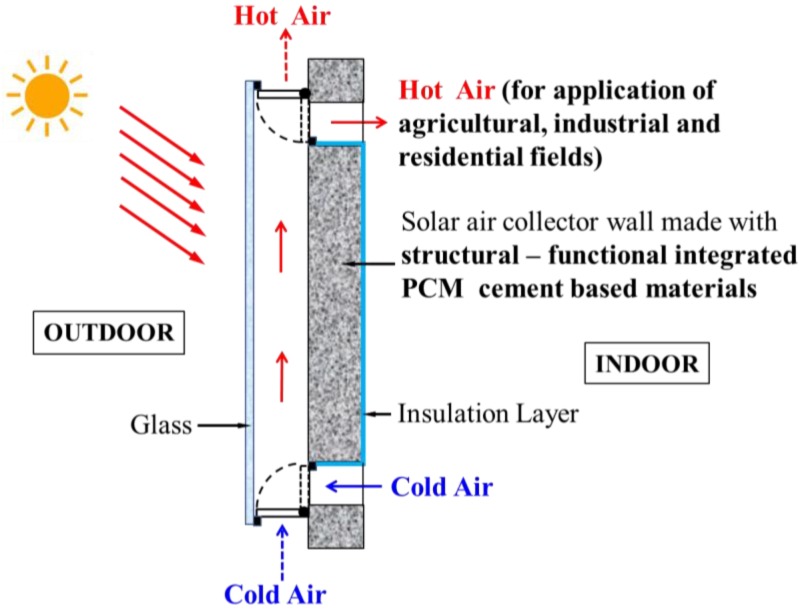
Schematic drawing of the solar air collector wall.

**Figure 2:**
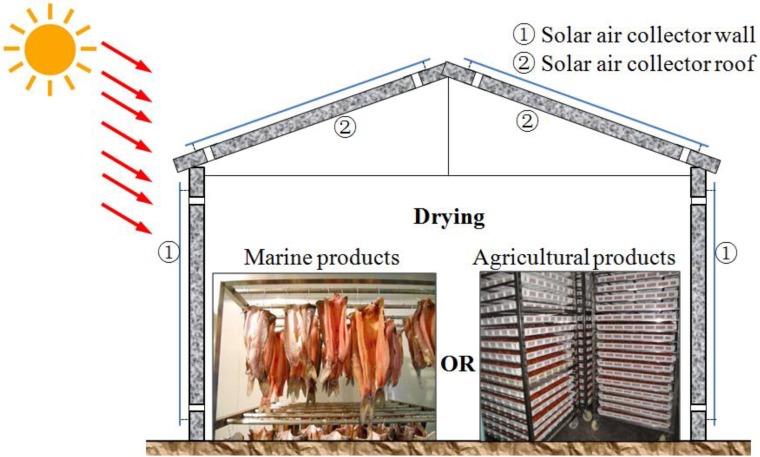
Schematic drawing of an instance of a building for applications of the solar air collector wall and roof.

Thermal applications are drawing increasing attention in the solar energy research field due to their high performance in energy storage density and energy conversion efficiency. In these applications, thermal energy storage systems and solar collectors are the two core components [7]. Among thermal energy storage techniques, the method of latent heat storage utilizing phase change materials (PCMs) has received significant attention among researchers and engineers due to its advantage of high energy storage density over a small temperature range. Phase change material (PCM) works on the principle that as the temperature increases, the material stores energy by changing its phase from solid to liquid within a defined temperature change, and then, when the temperature decreases, it releases heat by changing its phase from liquid to solid. When the phase change occurs, the temperature of the PCM remains almost constant. Applying PCMs to solar collectors installed in buildings, such as residential houses and greenhouses, is becoming more and more popular [8,9,10,11,12], and in these applications, the melting points of PCMs are required to be in the 50 to 70 °C range. In fact, up to now, most solar collectors have been separated from building walls or roofs. However, in order to improve the construction efficiency and for convenience, a precast wall (or roof) built-in solar air collector (solar air collector wall or roof) can be developed. In addition, PCM can be incorporated into construction materials by directly mixing the PCM with construction materials or by impregnating construction materials into the PCM [13]. If thermal energy storage cement paste (TESCP), a kind of structural-functional integrated PCM cement-based material, can be developed, then the material can be applied to cast building walls (or roofs) and solar air collectors with PCM more directly and more conveniently. Figure 1 shows the schematic drawing of the solar air collector wall suggested in this research, which is also a part of a building. The wall consists of covered glass, a structural-functional integrated PCM cement-based materials layer and an insulating layer. Through the solar air collector, the cold air can become hot, which can be used for many potential applications, such as for drying of agricultural, textile and marine products (PCM melting point: 50–70 °C) [12] and for heating of buildings to maintain a comfortable environment (PCM melting point: 25–30 °C) [14], especially in the winter season. Since PCM is added into the cement paste, the hot air can be supplied for a longer time compared with a conventional solar air collector. Moreover, for the solar air collector wall, the air vents can be opened or closed according to the seasonal requirements or the application aims of the hot air. Figure 2 shows an instance of a building for potential applications of the solar air collector wall and roof.

A structural-functional integrated cement-based material means that this material serves both as a structural material (having enough mechanical properties) for building applications and as a functional material (having thermal energy storage capacity). However, the major problem with this application is the leakage of PCM, especially after a number of thermal cycles. Furthermore, the mechanical and durability properties of the construction elements may be considerably affected. Hence, PCM must be encapsulated (macro- or micro-encapsulation), so that its application does not have a detrimental effect on the fundamental properties of the construction materials. However, macroencapsulation has the following disadvantages: (1) poor thermal conductivity; (2) it has to be protected against destruction (drilling holes or nails in the walls) while the building is in use [15]; (3) more on-site work is required for integration into the building structure [15]; and (4) an affinity towards solidification at the corners and edges, thereby preventing effective heat transfer. Therefore, microencapsulation, which provides a high heat transfer rate through its larger surface area per unit volume and which is capable of resisting volume change during phase transition, becomes the preferred choice.

Until now, few researchers have focuses on structural-functional integrated cement-based materials with microencapsulated phase change material (MPCM). Therefore, in this research, *in situ* polymerization, possibly the most feasible technique to encapsulate the liquid PCM, was used to prepare a MPCM with about a 55 °C melting point. For the development of structural-functional cement-based materials, the present research not only focused on the thermal properties of hardened cement paste with different mass percentages of MPCM, but also discussed their mechanical properties, which are comparatively less reported in the literature.

## 2. Materials and Experimental Methods

### 2.1. Materials

The following materials were used in this research: cement (Portland cement type 42.5R), paraffin (melting point of ~55 °C, supplied by Shanghai Yonghua Company, Shanghai, China), 37.0%–40.0% formaldehyde solution (analytical grade, supplied by Guangdong Guanghua Chimerical Plant Company, Guangdong, China), urea (analytical grade, supplied by Kaixin Chemical Reagent Company, Hengyang, Hunan, China), dodecyl benzene sulfonate solution (chemical grade, supplied by Tianjin Fuchen Chemical Reagent Company, Tianjin, China), 20% triethanolamine solution, 8% sulfuric acid solution and 1% sulfuric acid solution.

### 2.2. Preparation of Microencapsulated Paraffin

Microencapsulated paraffin was prepared using an *in situ* polymerization method. During the process of synthesis, formaldehyde solution and urea were used as synthetic materials for the shell, paraffin as the core material for the capsule, dodecyl benzene sulfonate solution as the dispersant and triethanolamine and sulfuric acid solutions to keep the pH value of the solution between 8 and 9. Since the temperature and stirring speed during the emulsification reaction and the dosage of paraffin and water would affect the shape, size and encapsulation of microencapsulated paraffin, after a trial-and-error procedure, the temperature during the preparation and the stirrer speed were obtained as 65 °C and 450 r/min, respectively. The dosage of formaldehyde solution and urea were maintained at 120 g and 60 g, while the dosage of paraffin and water were 280 g and 1200 mL, respectively.

**Figure 3:**
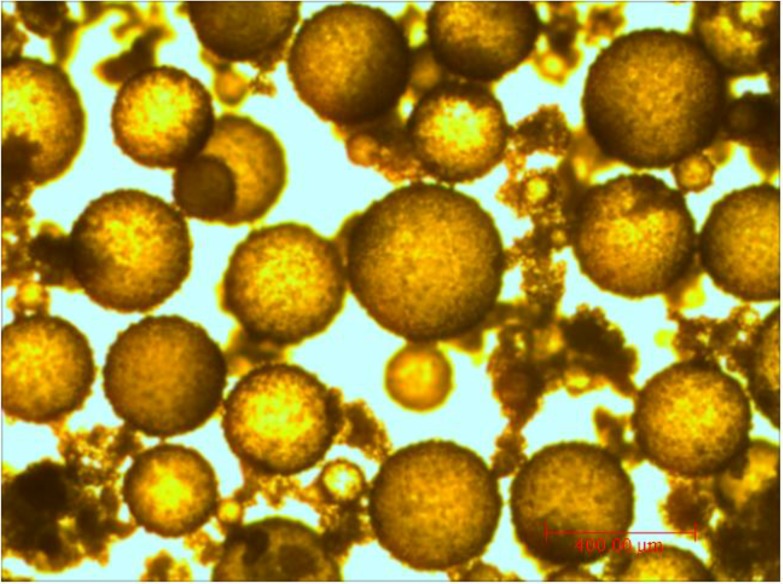
The morphology of newly-manufactured MPCM.

**Figure 4:**
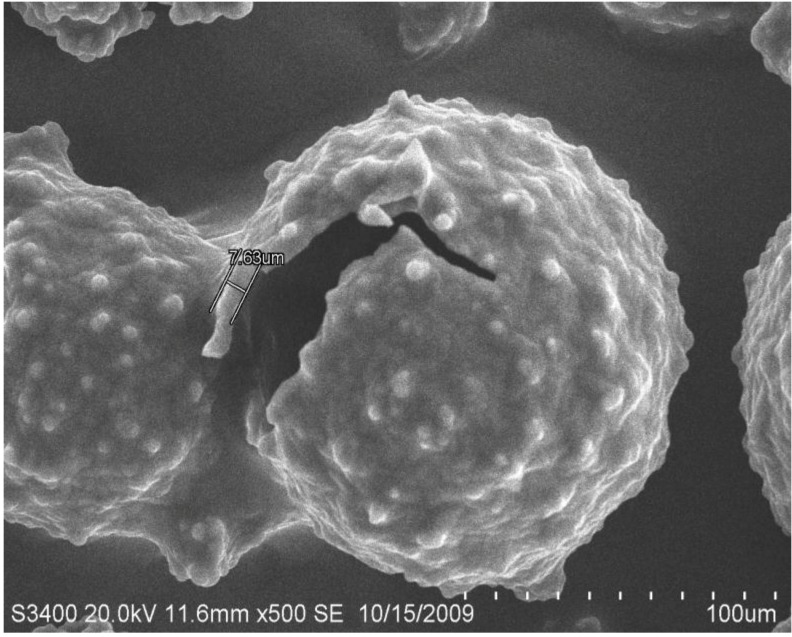
SEM image showing the MPCM shell thickness.

**Figure 5:**
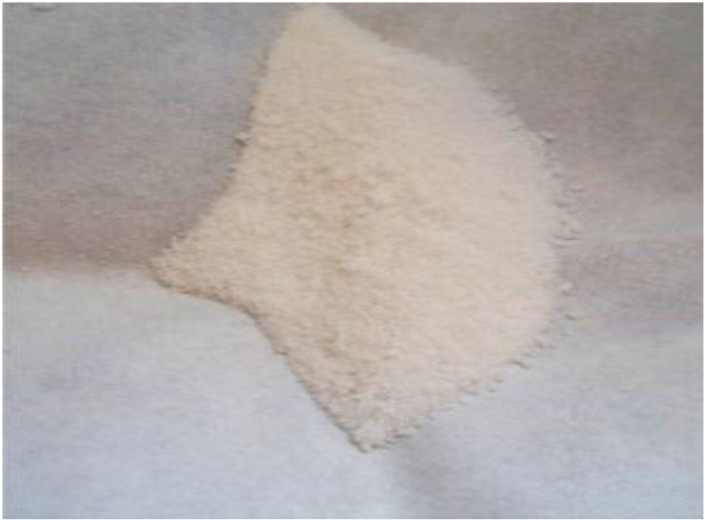
Microencapsulated paraffin in powder form.

The morphology of newly-manufactured MPCM examined by optical microcopy and SEM is shown in Figure 3 and Figure 4. It can be seen that the paraffin is encapsulated well (Figure 3), and the MPCM has a spherical shape. From Figure 4, the shell thickness of MPCM was determined as 7.63 μm. After the process of filtration and desiccation, the final form of the MPCM is shown in Figure 5, while the particle size distribution of MPCM, measured by a Mastersizer 2000 laser particle size analyzer (Malvern Instruments Ltd., Worcestershire, UK), is as follows: 

Particle size less than 200 µm = 11.7%

Particle size between 200 and 500 µm = 81.1%

Particle size more than 500 µm = 7.2%.

### 2.3. Preparation of Hardened Cement Paste Specimens with MPCM

#### 2.3.1. Mix Proportion

**Figure 6:**
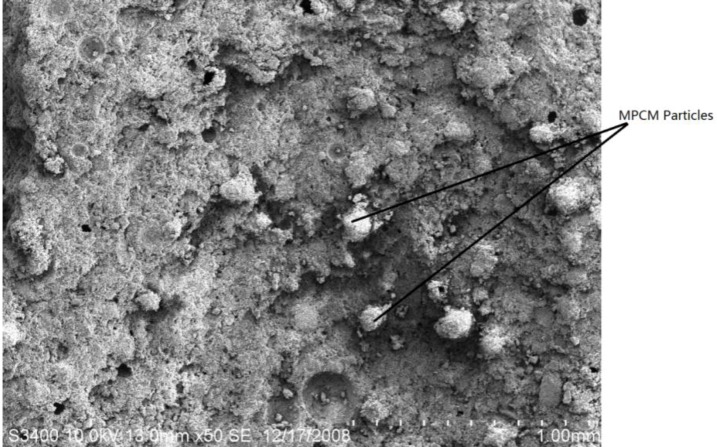
Microstructure of hardened cement paste with MPCM.

The details of the mix proportion of cement paste with a different mass percentage of MPCM are given in Table 1. As shown in Table 1, the mass percentages of MPCM were 0%, 5%, 10%, 15%, 20% and 25% by weight of cement, while the water cement ratio was 0.3. Moreover, from the microstructure of hardened cement paste (Figure 6), it can clearly be seen that the MPCM particles are undamaged during the mixing process.

**Table 1:** Mix proportion of harden cement paste with MPCM.

Mass ratio of MPCM by weight of cement	Cement	MPCM	Water
0 (control)	1	0	0.3
5%	1	0.05	0.3
10%	1	0.10	0.3
15%	1	0.15	0.3
20%	1	0.20	0.3
25%	1	0.25	0.3

#### 2.3.2. Cement Paste Mixing

MPCM and cement were firstly mixed at low speed for 1 min in a Hobart mixer (Hobart Corporation, Troy, OH, USA). Water was then added to the dry mixture, and constituents were mixed for 3 min at low speed and for another 1 min at high at speed. Thereafter, the fresh cement paste was cast in molds and compacted into three layers using a vibrating table. Finally, the cement paste specimens were tested after curing.

### 2.4. Test Methods

The thermal properties, *i.e.*, phase change temperatures and latent heat storage capacities, as well as the thermal stability, were determined using a thermal analyzer (STA409PC, NETZSCH, Selb, Germany) under a nitrogen atmosphere (20 mL·min^−1^) in the temperature range 30–75 °C and at a heating/cooling rate of 5 °C·min^−1^, which was adopted in other references [16,17,18]. At first, the temperature and heat flow calibrations were done using indium under nitrogen atmosphere. A known weight of sample was placed in a sealed aluminum pan for measurement, while an empty pan was used for reference. The thermal properties and thermal stability were determined using the Universal Analysis 2000 TA software package (TA Instruments, New Castle, DE, USA).

**Figure 7:**
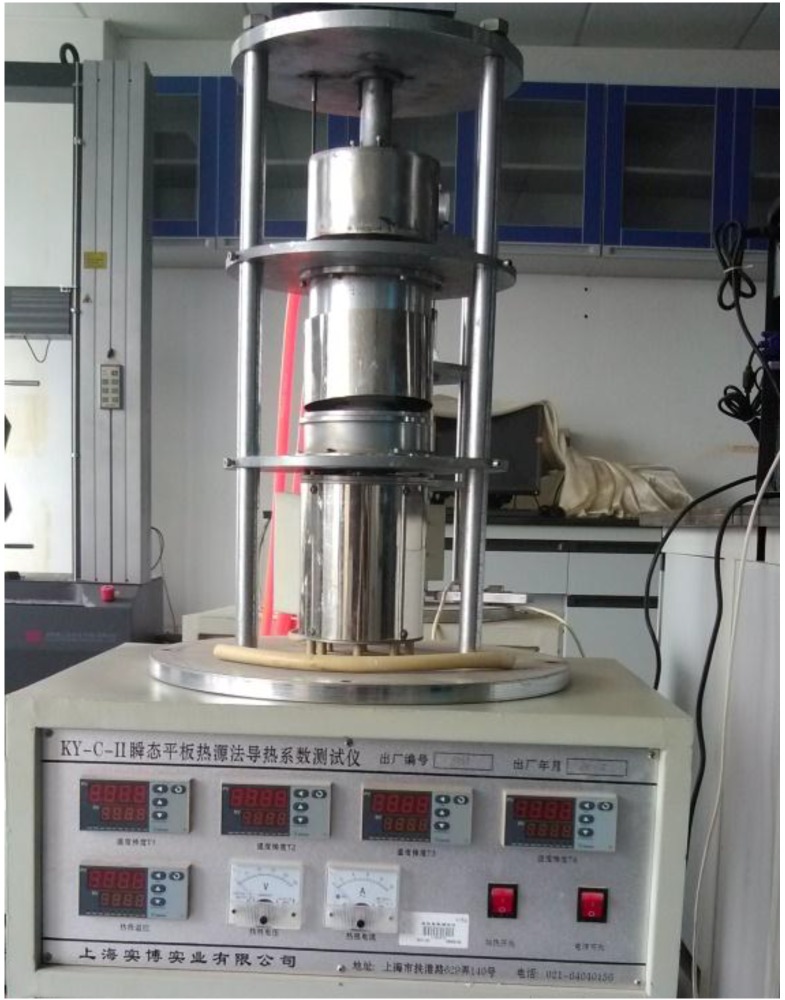
Thermal conductivity tester KY-C-II.

**Figure 8:**
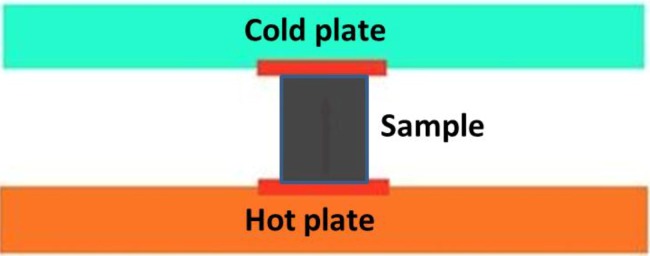
Schematic diagram of the thermal conductivity test.

The thermal conductivity of the cement paste disc prepared with and without MPCM was determined using the thermal conductivity tester shown in Figure 7 (Model Number KY-C-II, Shanghai Shibo Industrial Co., Ltd., Shanghai, China) complying with ASTM E1225-13 [19]. The samples having dimensions of 27 mm (diameter) × 6–8 mm (thickness) were tested at the age of 28 days. At first, the top and bottom surface of the plates were coated with thermally-conductive silicone, so as to have a good contact of the sample with the hot and cold surfaces of the tester (see Figure 8). The sample was then placed between the plates, and the heat flow through the sample was recorded when it reached a stable state. Thereafter, the thermal conductivity was calculated based on the heat flow readings. For this research, the thermal conductivity of the sample was determined by maintaining the temperature of the plates at the following three conditions.

(1) Temperature of the hot plate was 45 °C, while the temperature of the cold plate was set at 25 °C;
(2) Temperature of the hot plate was 65 °C, while the temperature of the cold plate was set at 45 °C;
(3) Temperature of the hot plate was 80 °C, while the temperature of the cold plate was set at 65 °C.

It needs to be pointed out here that the accuracy of the tester was ≤±3%. Three tests were done for each specimen at each temperature.

The compressive strength (40-mm cube) and flexural strength (40 mm × 40 mm × 160 mm prism) of the hardened cement paste was determined at the age of 28 days in accordance with GB/T 17671-1999 (method of testing cements-determination of strength) [20]. The loading rates for compressive and flexural strength were 2400 ± 200 N/s and 50 ± 10 N/s.

Before doing the compressive strength test, the dimensions of the sample were measured, and the mass of the sample was recorded using an electronic balance with a ±0.1 g error. The density of each sample was then calculated, which represented the average of three samples.

## 3. Results and Discussion

### 3.1. Phase Change Behavior and Thermal Reliability

Thermal gravimetric and differential thermal analysis (TG-DTA) was performed to quantitatively determine the phase change behavior and thermal reliability of paraffin, MPCM and cement paste containing different percentages of MPCM. The phase change behavior was determined in terms of phase change temperature and thermal energy storage, while the thermal reliability was determined by continuously monitoring the weight changes of the sample when subjected to repeated heating and cooling cycles at a controlled rate. In the following TG-DTA curves, the green curves represent TG curves, while the blue ones represent the DTA curve.

**Figure 9:**
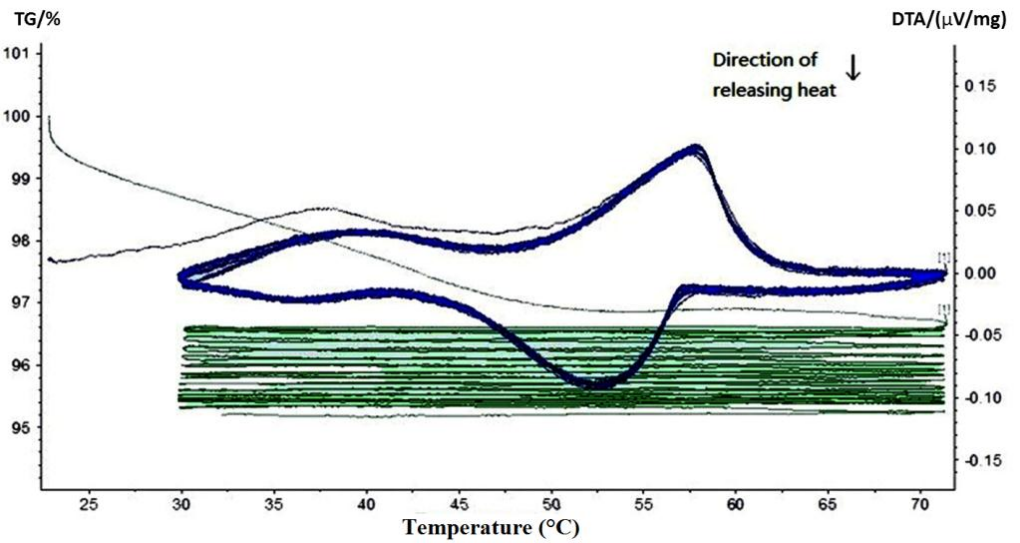
TG-DTA curve of the pure paraffin.

**Figure 10:**
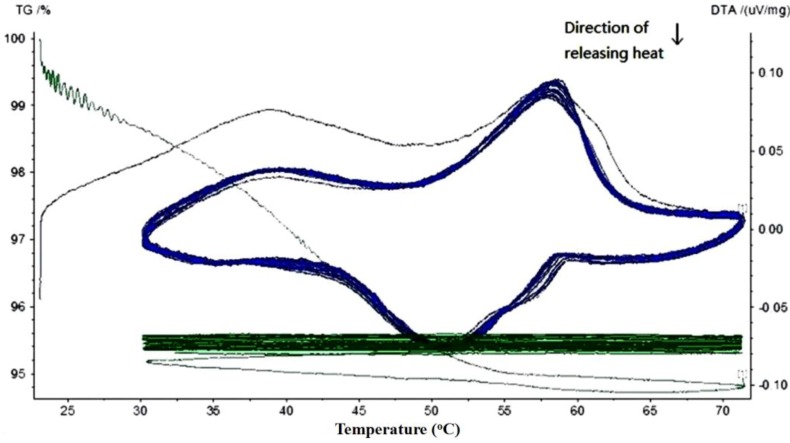
TG-DTA curve of the MPCM.

The TG-DTA curves of paraffin and MPCM are shown in Figure 9 and Figure 10, respectively. From DTA curves, using the Universal Analysis 2000 TA software package, the melting temperatures and latent heat of melting were determined as 49.9 °C and 222 J/g for paraffin and 49.9 °C and 202.71 J/g for MPCM. Moreover, the DTA curves of 15-times of heating and cooling cycles were nearly overlapped, which means that the MPCM maintained its heat absorption and release characteristics after 15 heating and cooling cycles, thus testifying to the thermal reliability of the MPCM and meeting the application requirement. From the experimental results, the encapsulation ratio of paraffin was calculated based on the enthalpy values using the following Equation (1) as 91.21 wt%.

(1) $$\text{η}(\text{\%})\;=\text{ Δ}{\text{H}}_{\text{MPCM}}/{\text{Δ}}_{\text{HPCM}}\;\times \;100\text{\%}$$

where η represents the mass percentage of paraffin in the MPCM and ΔH_MPCM_ and ΔH_PCM_ are measured latent heat of MPCM and pure paraffin, respectively.

The TG curves in Figure 9 and Figure 10 show a large slope during the first thermal cycle. Thereafter, the slope flattens with the increase in the number of heating and cooling cycles, indicating that the mass loss rate of MPCM decreases gradually to almost zero. The weight loss in paraffin and MPCM were found to be 4.8% and 4.4%, respectively. This minor difference means that the urea-formaldehyde resin wall of MPCM will remain stable under 30–75 °C. In fact, the working temperature range of the MPCM in this research also falls into 30–75 °C.

**Figure 11:**
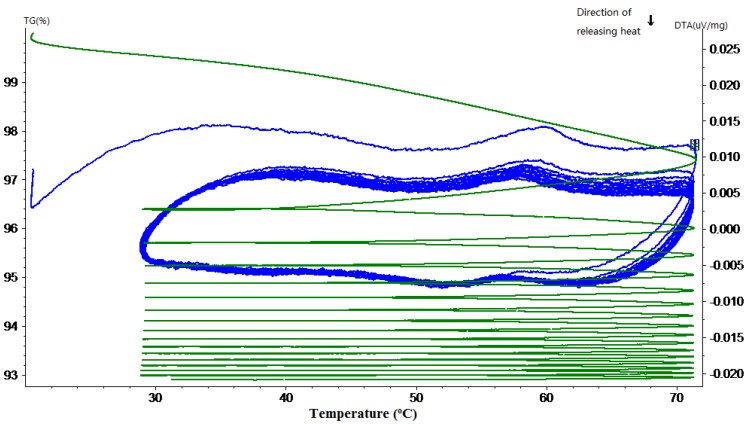
TG-DTA curve of the hardened cement paste specimen with 5% MPCM.

**Figure 12:**
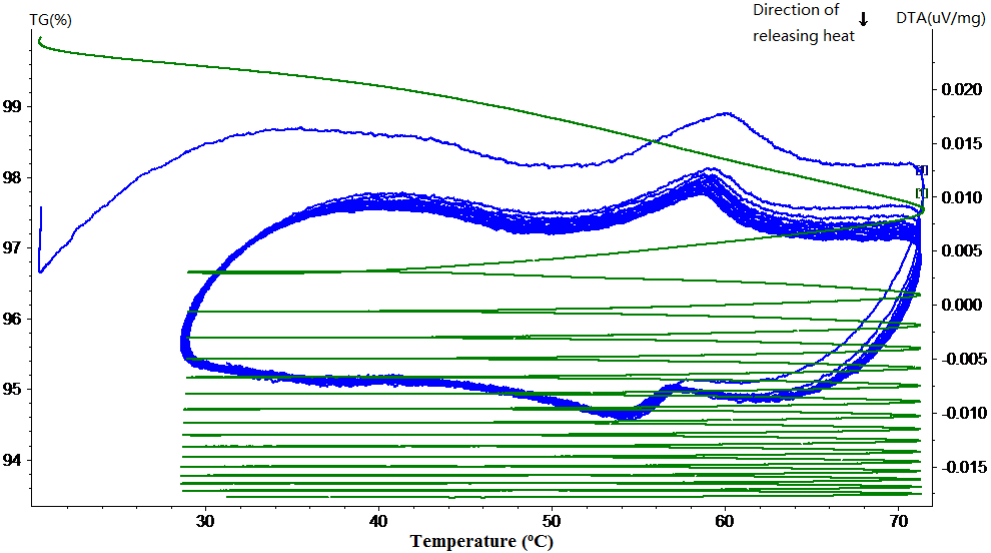
TG-DTA curve of the hardened cement paste specimen with 10% MPCM.

**Figure 13:**
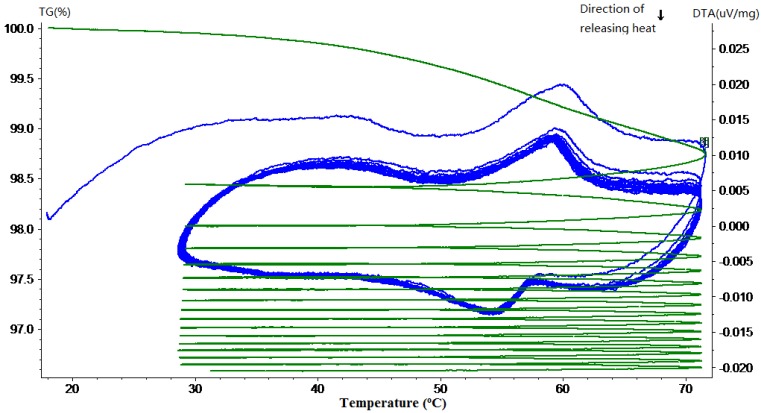
TG-DTA curve of the hardened cement paste specimen with 15% MPCM.

**Figure 14:**
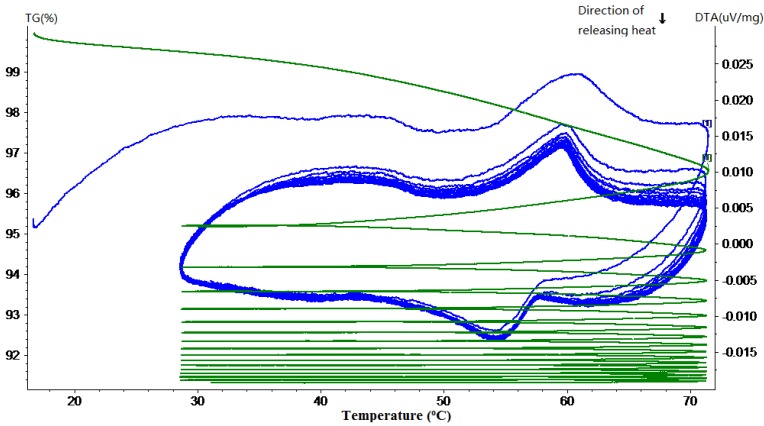
TG-DTA curve of the hardened cement paste specimen with 20% MPCM.

**Figure 15:**
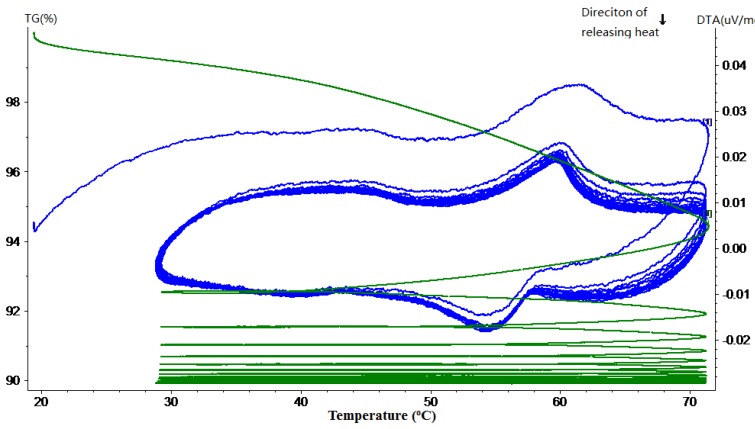
TG-DTA curve of the hardened cement paste specimen with 25% MPCM.

The TG-DTA curves of hardened cement paste with different mass percentages of MPCM are shown in Figure 11, Figure 12, Figure 13, Figure 14 and Figure 15. From these DTA curves, the values of latent heat of melting for the hardened TESCP specimens with 5%, 10%, 15%, 20% and 25% MPCM were found to be 7.52 J/g, 9.92 J/g, 14.10 J/g, 17.07 J/g and 20.47 J/g, respectively. Moreover, the phase change temperature range of TESCP specimens is 54~60 °C (*i.e.*, a 6 °C span). In order to visualize the enhancement of energy storage capacity with MPCM, the ratios of the latent heat of MPCM cement paste and the amount of heat needed to increase the temperature of ordinary cement paste by 6 °C are displayed in Table 2. The results show that the energy storage capacities of TESCP specimens with 5%, 10%, 15%, 20% and 25% MPCM are 1.4-, 1.9-, 2.7-, 3.2- and 3.9-times larger than that of ordinary hardened cement paste. This indicates that the energy storage capacities of TESCP specimens are significantly improved with the increase of MPCM content. Furthermore, MPCM in TESCP maintained its heat absorption and release characteristics even after 15 heating and cooling cycles according to the DTA curves of the hardened cement paste specimens with MPCM.

**Table 2:** Energy storage of specimens with different mass ratios of MPCM.

Latent heat of specimens	Mass ratio of MPCMs to cement (%)				
	5	10	15	20	25
^1^ Latent heat of cement paste specimens Q (kJ/kg)	7.52	9.92	14.10	17.07	20.47
^2^ Amount of heat needed to increase temperature of ordinary hardened cement paste by 6 °C (54–60 °C) (6 × Q_S_)	5.28	5.28	5.28	5.28	5.28
^2^ Q/(6 × Q_S_)	1.4	1.9	2.7	3.2	3.9

Notes: ^1^ size of test sample = 4 mm diameter × 3 mm height; ^2^ ordinary hardened cement paste’s specific heat capacity (Q_S_) = 0.88 kJ/(kg·°C) [21].

The TG curves in Figure 11, Figure 12, Figure 13, Figure 14 and Figure 15 show a large slope during the first thermal cycle, and then the slope flattens with an increase in the number of thermal cycles. Finally, the weight loss in cement paste specimens with 5%, 10%, 15%, 20% and 25% MPCM was found to be less than 7.5%, 7%, 6.5%, 9% and 10.5%, respectively, which are higher in comparison to the TG curves of the pure paraffin and the MPCM. This may be explained by the evaporation of water from the cement paste specimens. Moreover, since the DTA curves have a good repeatability and, meanwhile, the slope of TG curve gets close to zero, even after 15 thermal cycles, the MPCM has a good thermal reliability, and thus, the wall of MPCM can remain stable in the working temperature range.

### 3.2. Thermal Conductivity of Cement Paste with MPCM

**Figure 16:**
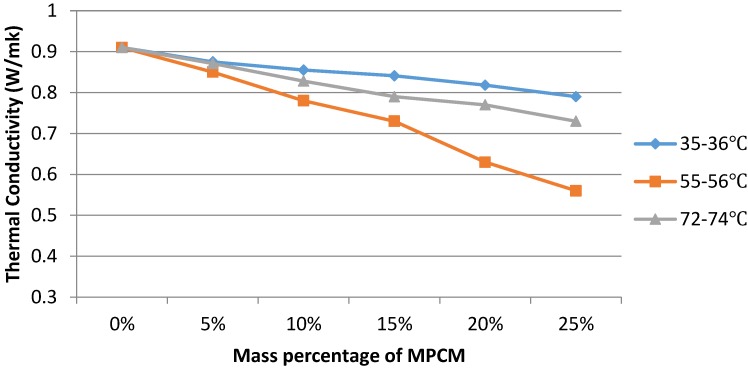
Thermal conductivity of hardened cement paste with MPCM at different average temperatures.

The thermal conductivity of PCM is one of the important parameters for latent heat thermal energy storage applications. The thermal conductivity of hardened cement paste was determined at the average temperatures of 35–36 °C, 55–56 °C and 72–74 °C. These average temperatures represent the condition of PCM in solid, phase transition range and liquid state. The test results of the thermal conductivity coefficient of hardened cement paste with different mass percentages of MPCM are presented in Figure 16.

When the average test temperature is 35–36 °C, the maximum temperature of the hot plate is around 45.9 °C, which is lower than the phase change temperature range of MPCM. Therefore, the PCM in the core of the microencapsulation does not reach the phase change temperature, *i.e.*, the paraffin is in the solid state. Because the thermal conductivity coefficient of solid paraffin is about 0.558 W·m^−^^1^·k^−^^1^, which is lower than the thermal conductivity coefficient of cement paste (0.912 W·m^−^^1^·k^−^^1^), the thermal conductivity decreases with the increase in the percentage of MPCM in hardened cement paste at this average temperature. In comparison to the control cement paste, the percentage reduction in thermal conductivity is 3.8%, 6.0%, 7.8%, 10.1% and 13.2% for 5%, 10%, 15%, 20% and 25% MPCM, respectively.

When the average test temperature is 72–74 °C, the maximum temperature of the hot plate is around 80.4 °C, while the minimum temperature of the cold plate is around 64 °C. This shows that the temperature in the specimen is above the phase transition temperature of the PCM. Therefore, the PCM in the core of the microencapsulation is in the liquid state. The thermal conductivity at this average temperature decreases with the increase in the percentage of MPCM in hardened cement paste. When the mass percentages of MPCM are 5%, 10%, 15%, 20% and 25%, the thermal conductivity of the cement paste reduces by 4.3%, 9.0%, 13.2%, 15.4% and 19.8%, respectively, greater than the reducing percentages when the average test temperature is 35–36 °C. This result is in line with the available literature [22], and it can be explained by the fact that paraffin in the solid state has a higher thermal conductivity than when in the liquid state.

When the average test temperature is near the phase transition temperature, *i.e.*, 55–56 °C, the maximum temperature of the hot plate is around 63.5 °C, while the minimum temperature of the cold plate is around 44.7 °C. The thermal conductivity at this average temperature decreases with the increase in the percentage of MPCM in hardened cement paste. The percentage reduction in thermal conductivity with 5%, 10%, 15%, 20% and 25% MPCM in the cement paste is 6.6%, 14.3%, 19.8%, 30.8% and 38.5%, respectively, greater than the reducing percentages when the average test temperatures are 35–36 °C and 72–74 °C. The reason for this phenomenon is that, when PCM has a phase change, the latent heat of the paraffin can store abundant heat. Therefore, in terms of the thermal conductivity coefficient, the values of the materials can be reduced obviously.

In previous studies, Fenollera *et al.* [23] also mixed self-compacting concrete (SCC) with different percentages of 0%, 5%, 10%, 15%, 20% and 25% MPCM by weight of cement, and the test results showed a linear relationship in which the thermal conductivity of SCC with MPCM decreases by 25% in the mix with 10% of MPCM by weight of cement. Besides, Hunger *et al.* [24] investigated the thermal conductivity of microencapsulated concrete when the temperature of the concrete sample was above the melting point of PCM and found that the addition of microcapsules resulted in the reduction of the thermal conductivity of concrete.

### 3.3. Compressive and Flexural Strength of Cement Paste with MPCM

**Figure 17:**
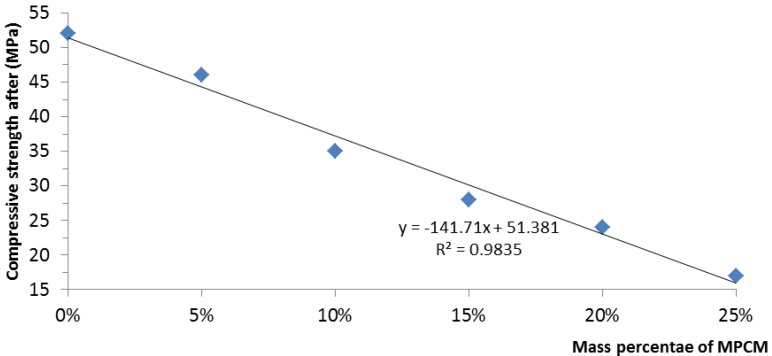
Relationship between the compressive strength and mass percentage of MPCM.

**Figure 18:**
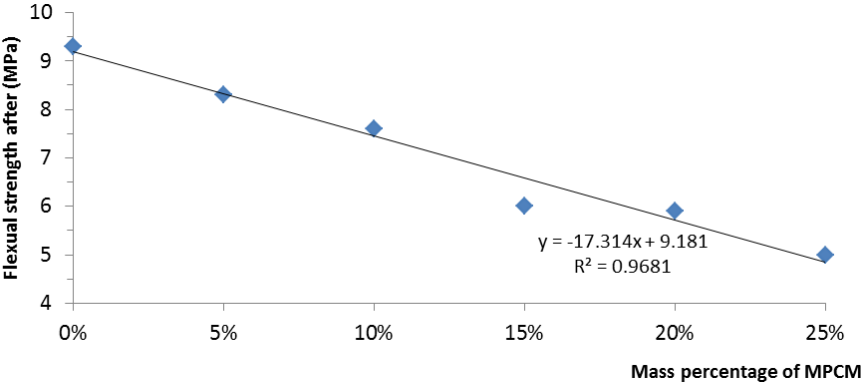
Relationship between the flexural strength and mass percentage of MPCM.

The results of the compressive strength and flexural strength of hardened cement paste with different mass percentages of MPCM are presented in Figure 17 and Figure 18.

As shown in Figure 17, the compressive strength of cement paste decreased with the increase in the mass percentage of MPCM. The decrease in compressive strength of the MPCM cement paste is due to the presence of weaker MPCM in compression. When the mass percentages of MPCM are 5%, 10%, 15%, 20%, and 25%, the reductions in compressive strength with respect to control cement paste are 11.5%, 32.69%, 48.07%, 55.77% and 66.34%, respectively. This shows that the maximum percentage reduction in compressive strength is between 5% and 10% MPCM. From Figure 17, it can also be seen that a linear relationship exists between the compressive strength and the mass percentage of MPCM. The regression relationship is expressed as follows,

(2) y = −141.71x + 51.381

where *y* is the compressive strength of the MPCM cement paste and *x* is the mass percentage of MPCM. The coefficient of determination *R*^2^ (0.9835) indicates the existence of a strong linear relationship between the compressive strength and the mass percentage of MPCM in the cement paste.

Figure 18 shows the results of the flexural strength of hardened cement paste with different mass percentages of MPCM. The flexural strength of cement paste with MPCM decreased with the increase in the mass percentage of MPCM. When the mass percentages of MPCM are 5%, 10%, 15%, 20% and 25%, the reduction in flexural strength with respect to control cement paste is 9.67%, 18.28%, 35.48%, 36.56% and 46.23%, respectively. This illustrates that the maximum percentage reduction in flexural strength is between 10% and 15% MPCM, while the percentage reduction in flexural strength was almost the same, between 15% and 20% of MPCM by weight of cement. It can also be witnessed that for a constant percentage of MPCM, the percentage decrease in compressive strength is more than the corresponding percentage decrease in flexural strength. In addition, the relationship between the flexural strength and the mass percentage of MPCM was found to be linear and is given by:

(3) y = −17.314x + 9.181

where *y* is the flexural strength of the MPCM cement paste and *x* is the mass percentage of MPCM. The value of the coefficient of determination *R*^2^ for this equation was found to be 0.9681, which indicates the existence of an almost linear relationship between the flexural strength and the mass percentage of MPCM in the cement paste.

Moreover, the significant detrimental effect of MPCM on the mechanical strength of the cement-based matrix has been observed by many previous studies. In [23], self-compacting concrete (SCC) was also mixed with different percentages of 0%, 5%, 10%, 15%, 20% and 25% MPCM by weight of cement, and the test results found a linear relationship for which the compressive strength of SCC with MPCM decreases by 7% for every 5% of added PCM. Besides, Eddhahak *et al.* [25,26] indicated a 47% reduction (from 36 to 19 MPa) for mortar and a 32% reduction (from 25 to 17 MPa) for concrete when 5% of MPCM with regard to concrete total volume was added into the cement-based matrix. Hunger *et al.* [24] experimentally investigated the effect of the incorporation of different percentages of MPCM (1%, 3%, 5% by weight of concrete total mass) on the compressive strength of concrete and found that the inclusion of 5% MPCM reduced the compressive strength by up to 69%.

### 3.4. Density of MPCM Cement Paste

**Figure 19:**
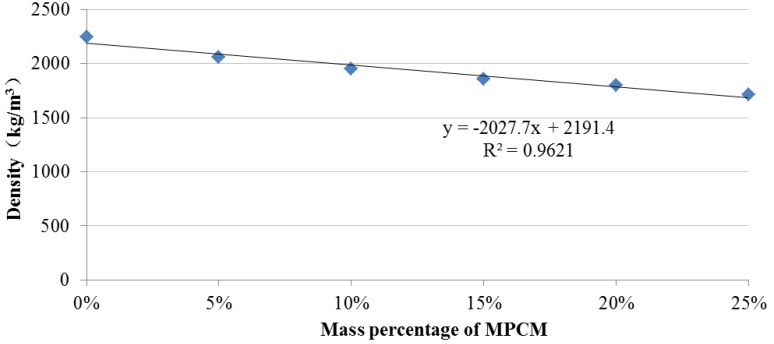
Density of hardened cement paste with different mass percentages of MPCM.

The results of the density of cement paste containing MPCM with its mass percentage varying from 0% to 25% by weight of cement are shown in Figure 19. The density of cement paste decreased with the increase in the mass percentage of MPCM, which is in accordance with the result of [24]. The decrease in density can be attributed to the low density of PCM (0.78/cm^3^) and the structural change in cement paste packing due to the introduction of MPCM. When the mass percentages of MPCM are 5%, 10%, 15%, 20% and 25%, the decreases in density are 8%, 13%, 17%, 20% and 23%, respectively. Figure 19 shows that a strong linear relationship between density and mass percentage of MPCM is found as follows:

(4) y = −2027.7x + 2191.4

where *y* is the density of MPCM cement paste and *x* is the percentage of MPCM. The coefficient of determination *R*^2^ (0.9621) indicated the existence of a strong linear relationship between the density and mass percentage of MPCM in the cement paste. Similarly, Fenollera *et al.* [23] reported that the density of fresh SCC with MPCM decreases by 1.1% per every 5% of added PCM by weight of cement, according to the densities of fresh SCC with different percentage of 0%, 5%, 10%, 15%, 20% and 25% MPCM.

## 4. Conclusions

From the experimental investigation, the following conclusions can be drawn:

- The prepared MPCM has a melting temperature of 49.9 °C and a latent heat of 202.71 J/g. The encapsulation ratio of paraffin was found to be 91.21 wt%. Moreover, microencapsulation improved the thermal reliability of paraffin.
- The thermal energy storage capacity of cement paste with 25% MPCM by weight of cement was found to be 3.9-times higher than that of cement paste alone.
- The thermal conductivities of hardened cement paste determined at the average temperatures of 35–36 °C, 55–56 °C and 72–74 °C decreased with the increase in the percentage of MPCM in hardened cement paste. The maximum percentage reductions at the average temperatures of 35–36 °C, 55–56 °C and 72–74 °C were found to be 10.1%, 38.5% and 19.8%, respectively. Furthermore, for a constant percentage of MPCM, the thermal conductivity of the hardened cement paste decreased with the increase in the average test temperature.
- The compressive strength, flexural strength and density of hardened cement paste with MPCM decreased with the increase in the content of MPCM in cement paste, and they had a strong linear relationship. Moreover, compressive strength had a larger and faster degradation with the increase of MPCM content among them.
